# COVID-19 status and utilisation of essential maternal and child healthcare services during the pandemic in Ahmedabad, India

**DOI:** 10.1186/s12884-025-07201-2

**Published:** 2025-01-30

**Authors:** Sandul Yasobant, K Shruti Lekha, Ravina Tadvi, Bhavin Solanki, Walter Bruchhausen, Deepak Saxena

**Affiliations:** 1 Department of Public Health Science, Indian Institute of Public Health Gandhinagar (IIPHG), Gandhinagar, Gujarat India; 2 Centre for One Health Education, Research & Development (COHERD), Indian Institute of Public Health Gandhinagar (IIPHG), Opp. Air Force Head Quarters, Nr. Lekawada, Gandhinagar, 382042 Gujarat India; 3 School of Epidemiology & Public Health, Datta Meghe Institute of Medical Sciences (DMIMS), Wardha, India; 4 Global Health, Institute for Hygiene & Public Health, University Hospital Bonn, Bonn, Germany; 5 Department of Health, Ahmedabad Municipal Corporation (AMC), Ahmedabad, India

**Keywords:** COVID-19 pandemic, Maternal & child healthcare services, Disruption, Healthcare service utilisation, Ahmedabad, India

## Abstract

**Background:**

Access to essential healthcare services is pertinent to the achievement of universal health coverage in any nation. The COVID-19 lockdown was used to mitigate the spread of the pandemic. Consequently, there was a reduction in the Utilisation of Basic Healthcare Services (UBHS) in diverse dimensions. However, variation existed in the UBHS by COVID-19 status, but the extent of this disparity has not been extensively addressed in Ahmedabad, India. Therefore, this study explores the relationship between COVID-19 status and utilisation of essential maternal and child healthcare services during the pandemic in Ahmedabad, India.

**Methods:**

A mixed-method approach was used for the data (both quantitative and qualitative) collection from November 2021 to October 2022. Four zones were purposefully selected from the 6 zones in Ahmedabad. The quantitative part of the study included pregnant women or those who had a baby delivery from April 2020 to October 2021 (*n* = 278), while 10 of these women participated in the qualitative part. Data were analysed using descriptive statistics, Chi-square test, and binomial logistic regression (α = 0.05). A deductive approach was used to analyse the qualitative data.

**Results:**

Of the total 278, almost 43% of the women were infected with COVID-19 during their pregnancy. Women who tested positive availed lesser antenatal care (ANC), and Postnatal care (PNC). There were diverse experiences documented regarding access to essential maternal and child healthcare services during the pandemic. Women without COVID-19 are more likely to receive maternal healthcare services, such as visits of any healthcare workers [aOR = 2.59 (1.03- 6.49)], counseling services [aOR = 1.92 (0.61- 6.06)], delivery at the planned place [aOR = 1.98 (0.99- 3.92)] as compared to those who are positive. Women without COVID-19 were more likely to be accompanied by healthcare workers during labor ([aOR = 2.91(1.04- 8.11) and to receive appropriate birth spacing counselling [aOR = 1.38 (0.7–2.71)].

**Conclusion:**

Utilisation of essential maternal and child healthcare services was lower among women who were COVID-19 positive compared to those who were not. Social and health system determinants for disrupting healthcare services during the pandemic were fear of infection and unavailability of the health workforce. Health planners and policymakers are encouraged to take into consideration of these findings while building resilient health care for managing future pandemics.

## Introduction

The COVID-19 pandemic has not only led to compulsory lockdowns, curfews, and containment of activity in societies [1] but also reduced the utilisation of healthcare facilities, especially among individuals with less severe illnesses [2, 3]. Many private hospitals and health centres ended up providing emergency services only and not engaging with routine patients [4]. World Health Organization has also stated, "People, efforts, and medical supplies all shift to respond to the emergency”; this often leads to the neglect of basic and regular essential health services [5]. People with health problems unrelated to the epidemic find it harder to access healthcare services during the pandemic [6], which results in reduced essential healthcare availability and accessibility.

The health systems framework assumes that four components affect the coverage of services: availability of health workers, availability of supplies and equipment, demand for services, and access to services [4]. A change in demand or ability to provide health services during emergencies can disrupt routine and essential health services such as immunization. When disruptions are not anticipated and mitigated, there can be excess morbidity and mortality due to a lack of routine health services [7]. This could be an additional burden during the pandemic.

In India, among others, maternal and child health services are considered as one of the essential routine healthcare services. The National Family Health Survey (NFHS)−5, which is a nationwide survey conducted during 2019–2020, reported that 82.4% of women went for 3 or more Antenatal care checkups (ANC), 97.8% of women had institutional deliveries or delivery by trained personnel, 91.4% of women received 2 doses of TT and 93.1% women received follow up visits post-delivery. There was no further documentation on how the pandemic disrupted these essential services. The Ebola (EVD) outbreak in West Africa of 2014–2015 is a classic example that created additional delays in the ANC and PNC services. The famous five-delay model emerged during these testing situations [8, 9]. The emergencies result in a shortage of medications due to disruption in supply chains as well as the shortage of resources. On the demand side, fear and uncertainty decrease the demand and utilisation of healthcare. During the African EVD outbreak, the utilisation of health services declined significantly. The experience of fatalities led to beliefs that health facilities should be avoided. The Ebola crisis taught a valuable lesson about what happens when an outbreak takes health workers away from core functions to focus on crisis response; the number of people who died from reduced access to usual care probably exceeded the number killed by the virus [10]. The nationwide lockdown during the COVID-19 pandemic might have severe implications for maternal and child healthcare services [5, 11, 12].

One study from India reported that the COVID-19 pandemic substantially affected maternal and perinatal healthcare service delivery and utilization [13]. This is a severe setback also to the Sustainable Development Goals (SDG)−3 goal of target 1 aims to reduce global maternal mortality. To achieve this, it is essential to ensure uninterrupted maternal care services during the COVID-19 pandemic [12]. It has also been seen that there has been a decline in utilization of services mainly the essential services in China [14]. Primary health care and specialty care both increased after COVID-19 diagnosis similarly, visits to specialists also significantly increased post testing positive [15]. Similarly, the patients who were tested positive for COVID-19 seek frequent health care and also have a higher demand for the health care [16]. Therefore, this study aims to document the utilisation of essential maternal and child healthcare services during the COVID-19 pandemic in Ahmedabad, India. It estimated the proportion of utilisation of health services by pregnant women during the pandemic (from the quantitative aspect). It also sheds light on the reasons for not availing or availing Antenatal Care (ANC), Intranatal Care (INC), Post Natal Care (PNC), and child healthcare services during the pandemic and a few social and health system determinants for the disruption of healthcare services during the pandemic. It also explored the experiences of the women while accessing maternal and child healthcare services during the pandemic (from the qualitative aspect). The key hypothesis was women who were COVID-19-negative during their pregnancy had higher healthcare access as compared to the women who were positive in Ahmedabad, India.

## Methods

The mixed-method approach was carried out in Ahmedabad, Gujarat, India. The study was conducted from November 2021 to October 2022.

### Study setting

Ahmedabad is a city in the west of India. It is the 5th most populous city in the country and the most populous city in Gujarat. The Forbes list ranked it the third fastest-growing city of the decade in 2010. The whole city of Ahmedabad has been divided into 6 Administrative divisions: Central, East, West, North, South, and North West zones. Ahmedabad is managed by a corporate body called Ahmedabad Municipal Corporation (AMC). The health department of AMC is responsible for managing the city's health system.

Ahmedabad, a city with a rich historical past, has faced and overcome significant public health challenges. It had a history of several outbreaks in the recent past, like Crimean-Congo hemorrhagic fever (CCHF), Avian influenza (H5N1), Rabies, chikungunya, and zika, among others. Evidence of the city's ability to manage epidemics throughout its history provides a strong foundation for addressing contemporary healthcare needs. However, the city's rapidly growing population presents a significant challenge in ensuring all its residents' access to quality healthcare services.

### Study design

It is a mixed-method study involving quantitative and qualitative data collection methods from April to June 2022. The quantitative data was collected through a cross-sectional survey, whereas the qualitative data was collected through the narrative study using in-depth interviews. The quantitative arm estimated mothers' utilization of ANC, INC, PNC, and immunization services during the pandemic and child healthcare services. A cross-sectional survey among pregnant women or delivered during the pandemic was conducted to gather information on the utilization of maternal and child healthcare services. The qualitative arm was carried out to explore the experience of using maternal and child healthcare services during the pandemic through narrative study. In-depth interviews of pregnant women who delivered a baby during the pandemic were conducted to document their experiences.

### Study samples and sampling

Four zones were purposefully selected from the 6 zones in Ahmedabad. In the first stage, each zone was considered a cluster. About 04 healthcare facilities were randomly selected in the second stage from each cluster to represent the city with consideration of the diversity. In the third stage, out of all eligible women per healthcare facility who provided consent, they were recruited for the study. Fifty women per healthcare facility per cluster was the initial targeted sample size. Women who had their delivery at a government or a private facility during the pandemic and were residing within the city were included in the study, whereas the women who didn’t deliver due to abortion or had a miscarriage or migrated were excluded from the study.

#### Quantitative assessment

The quantitative assessment was carried out among women who were pregnant or had delivered during the pandemic. The women who provided consent were selected from the total list of eligible women who delivered or were pregnant from April 2020 to October 2021. About 278 women were recruited for the quantitative assessment, irrespective of their COVID-19 infection status.

#### Qualitative assessment

IDIs were carried out among ten mothers who had delivered during the pandemic, irrespective of their COVID-19 infection status. The mothers were selected purposively to document the diverse delivery experiences.

#### Operational definitions

1. COVID-19-positive mothers: The women who were pregnant or delivered a baby during the pandemic but were exposed to the Sars-CoV2 virus and tested positive during their pregnancy.
2. COVID-19-negative mothers: The women who were pregnant or delivered a baby during the pandemic but never tested positive for the Sars-Cov2 virus during their pregnancy.

### Data collection

#### Quantitative data collection

The quantitative information was collected using a structured survey questionnaire. The information sought concerned such data as basic demographic details about ANC and PNC services and reasons for not availing of the services The details like demographic details, details on reproductive history like the parity, gravida, type of delivery, place of delivery, services that were availed as part of ANC, INC, and PNC, services availed during childcare, and reasons for not availing were explored and included in the data collection tool. The data were collected in the vernacular language by the trained researchers.

#### Qualitative data collection

The qualitative information was collected using a semi-structured interview guide by the trained researchers in the vernacular language. The semi-structured interview guide was designed to capture detailed information about their experiences during pregnancy, issues, and challenges faced during the delivery due to the pandemic, and issues in obtaining PNC and child healthcare services. Each interview was for about 30 to 45 min.

### Data analysis

The quantitative and qualitative data were analysed separately and triangulated for interpretation. The following procedures were used for the analysis.

#### Quantitative analysis

Once the data was collected, data sets were prepared and validated in Microsoft Excel. Following that, data was cleaned and analysed using the statistical software STATA version 14.1.

- Dependent Variable: Status of COVID-19 (COVID -ve mothers vs COVID + ve mothers)
- Independent Variable: Socio-demographic factors, Maternal healthcare (ANC, INC, and PNC) services, and Child healthcare services

A descriptive analysis of the basic socio-demographic details was carried out. The bivariate analysis was out conducted to assess the difference in utilization of ANC, INC, PNC, and child healthcare services during the pandemic between women who tested positive for COVID-19 and those who didn't test positive for COVID-19. A chi-square test was conducted to observe the statistical significance. Binomial logistic regression analysis was performed to evaluate the differential utilization of these services in relation to their status of COVID-19. The COVID-19-negative mothers were considered as an outcome of interest as compared to the positive mothers to understand the reach of healthcare accessibility.

#### Qualitative analysis

Firstly, all the recorded audio was transcribed and then translated into English. Then, the thematic analysis was done, and the data was coded based on that. The data analysis involved various team members to ensure reliability, reproducibility, and stability. A deductive approach was used to understand the healthcare availability and accessibility challenges experienced by the recruited women. In the result section, significant quotes were shown under two prime thematic domains, i.e., social and health system determinants for the disparity in the utilisation of services during the pandemic.

## Results

### Quantitative findings

A total of 278 women were recruited for the study. Of the total 278, almost 43% of the women were infected with COVID-19 during their pregnancy. Among the study participants, the majority (87.4%) were Hindus, 48.6% belonged to “other backward castes” (OBCs). As shown in Table 1, the age of the participants ranged from 18 to 42 years, with the mean age of the participants being 27.5 years. Almost one-fourth of the participants were below 24 years of age. Only 8% of women had not received any formal education. 12.2% belonged to a below-poverty-line family. 8% of women had a history of any pre-existing chronic illness, hypertension, or hypothyroidism. Of the total, 34.5% were nulliparous.

**Table 1:** Basic socio-demographic details of mothers recruited during November 2021- October 2022 (*n* = 278)

Characteristics	Number (%)
*Status of COVID19*	
No	159 (57.2)
Yes	119 (42.8)
*Religion*	
Hindu	243 (87.4)
Muslim and Christians	35 (12.6)
*Caste*	
General	102 (36.7)
OBC^*^	135 (48.6)
SC & ST	41 (14.7)
Age Group	
Below 24 years	73 (26.3)
24–27 years	76 (27.3)
28–31 years	73 (26.3)
Above 31 years	56 (20.1)
*Education*	
No formal education	22 (7.9)
Primary	69 (24.8)
Secondary	83 (29.9)
Higher Secondary	36 (12.9)
Graduate	47 (16.9)
Postgraduate and above	21 (7.6)
*Family Economic Status*	
APL	169 (60.8)
BPL	34 (12.2)
Don't know	75 (27)
*History of pre-existing chronic diseases and risk conditions*	
No	255 (91.7)
Yes	23 (8.3)
*Natality Status of Gravida*	
Nulliparous	96 (34.5)
Multiparous	182 (65.5)

OBC* Other Backward Caste, *SC* Schedule Caste, *ST* Schedule Tribe, *APL* Above Poverty Line, *BPL* Below Poverty Line

^*^Other Backward Classes means any socially and educationally backward classes of citizens as declared by the Government of India/respective state

### Maternal healthcare services

ANC services are an integral and essential part of the health system. It is a cluster of services provided to a woman during the pregnancy aiming to look after the mother's health or her unborn baby. In the study, a total of 88.5% of mothers were registered during the pregnancy. Out of COVID-negative mothers, 96.9% were registered during pregnancy, while among COVID-positive mothers, only 77.3% were registered. Almost half (48.7%) of the COVID-positive mothers had visited a private facility for an ANC check-up. In comparison, among the COVID-negative mothers, More than fifty percent of mothers (52.2%) had visited a government hospital. A health worker at the doorstep had visited around 91% of the COVID-negative mothers, whereas only 63% of COVID-positive mothers had been visited by a health worker. The details of the other services provided are in Table 2, a significant association between the COVID-19 status of the mother and registration of pregnancy (*p* = 0.000), visit the hospital for their ANC check-up (*p* = 0.010), Place of ANC check-up (*p* = 0.022), visit by ASHA or CHWs (0.000), Receiving mother and child protection card (*p* = 0.000), made aware about subsequent antenatal visits or expected date of delivery (*p* = 0.006), received iron-folic acid and calcium supplements (*p* = 0.010), received counselling related to increase consumption of iron-rich food source (*p* = 0.014), discuss or prepare a birth plan for pregnancy (*p* = 0.000) as seen in Table 2.

**Table 2:** Details of ANC services availed by mothers stratified with their COVID-19 status during November 2021- October 2022 in Ahmedabad, India

Maternal Health Services	Total *N* = 278 (%)	COVID -ve mothers *n* = 159 (%)	COVID + ve mothers *n* = 119 (%)	*p*-Value
Registration of pregnancy by CHW	246 (88.5)	154 (96.9)	92 (77.3)	0.000
Optimal ANC	270 (97.1)	158 (99.4)	112 (94.1)	0.010
Place of ANC check-up				
Government facility	127(45.7)	83 (52.2)	44 (37)	0.022
Private facility	115 (41.4)	57 (35.9)	58 (48.7)	
Both	36 (12.9)	19 (11.9)	17 (14.3)	
Door step visit of healthcare workers	219 (78.8)	144 (90.6)	75 (63.0)	0.000
Recipient of Mother and Child Protection Card	245 (88.1)	153 (96.2)	92 (77.3)	0.000
Awareness of EDD	248 (89.2)	149 (93.7)	99 (83.2)	0.006
Recipient of iron-folic acid and calcium	236 (84.9)	143 (89.9)	93 (78.2)	0.007
High Risk pregnancy	25(9)	12 (7.5)	13 (10.9)	0.032
Recipient of counselling services	239 (86)	144 (90.6)	95 (79.8)	0.011
Optimal birth plan	184 (66.2)	126 (79.2)	58 (48.7)	0.000
Enrollment into the government scheme (JSY, JSSK, KPSY, PMSMA or SUMAN)	228 (82)	126 (79.2)	102 (85.7)	0.027

*CHW* Community Health Worker, *ASHA* Accredited Social Health Activist, *ANM* Auxiliary Nurse and Midwife, *EDD* Expected Date of Delivery, *JSY* Janani SurakshaYojana, *JSSK* Janani Shishu Suraksha Karyakaram, *KPSY* Kasturba Poshan Sahay Yojana, *PMSMA* Pradhan Mantri Surakshit Matritva Abhiyan, *SUMAN* Surakshit Matritva Aashwasan

The data shows that most mothers delivered at a public facility irrespective of their COVID-19 status; however, more mothers tested positive delivered at public facilities than negative mothers. Most mothers (78.1%) preferred their own or private vehicles to reach the hospital during pregnancy. For most (82.4%) of the COVID-negative mothers, the actual place of delivery was the same as planned, while for only 58% of the COVID-positive mothers. There is a significant association between COVID-19 status and change in the planned place of delivery (*p* = 0.000), the person accompanying during delivery (*p* = 0.026), and received counselling related to birth spacing/ planning (*p* = 0.004) as seen in Table 3.

**Table 3:** Details of INC and PNC services availed by mothers stratified with their COVID-19 status during November 2021- October 2022 in Ahmedabad, India

Maternal Health Services	Total *N* = 278 (%)	COVID -ve mothers *n* = 159 (%)	COVID + ve mothers *n* = 119 (%)	*p*-Value
**Place of delivery**				
Public facility	149 (53.6)	79 (49.7)	70 (58.8)	0.084
Private facility	122 (43.9)	73 (45.9)	49 (41.2)	
Home	2 (0.7)	2 (1.3)	0 (0.0	
Trust hospitals	5 (1.8)	5 (3.1)	0 (0.0)	
**Type of transportation used to reach hospital during labor/pregnancy**				
108	59 (21.2)	29 (18.2)	30 (25.2)	0.000
Own/Private	217 (78.1)	128 (80.5)	89 (74.8)	
No use of transport	2 (0.7)	2 (1.3)	0 (0.0)	
**Planned place of delivery same as the actual place of delivery**				
No	78(28)	28(18.5)	50(42)	0.000
Yes	200(72)	131(81.5)	69 (58)	
**The person who conducted the delivery**				
Appointed medical officer	258 (92.8)	147 (92.5)	111 (93.3)	0.467
Nurse/ANM	18 (6.5)	10 (6.3)	8 (6.7)	
Untrained staff	2 (0.7)	2 (1.3)	0 (0.0)	
**Had anyone accompany you during your labor**				
No	27 (9.7)	10 (6.3)	17 (14.3)	0.026
Yes	251 (89.9)	149 (93.7)	102 (85.7)	
**Received any counseling related to birth spacing/family planning**				
No	97 (34.9)	44 (27.7)	53 (44.5)	0.004
Yes	181 (65.1)	115 (72.3)	66 (55.5)	

*ANM* Auxiliary Nurse and Midwife

Vaccination services are an essential aspect of children's and maternal health. Almost 97% of COVID-negative mothers received two doses of the Tetanus Toxoid (TT) vaccine, while 92% of the COVID-positive mothers received two doses of the TT vaccine. Of the children, 84.3% received immunisation per age among the COVID-negative mothers and 77% among the COVID-positive mothers, a difference found to be statistically significant (*p* = 0.003).

Child healthcare services are crucial for the health of the child. Of the total, 92.8% received vitamin K injections among COVID-negative mothers, while only 87% among COVID-positive mothers. Around 61% of children of COVID-negative mothers, while only 44% of children of COVID-positive mothers were provided with Iron and folic acid supplements. The findings show that there is a statistical association between COVID-19 negative status of the mother and the baby receiving vitamin K injection after birth (*p* = 0.001), child provided with IFA (*p* = 0.000), child provided with ORS packets (*p* = 0.000), child provided with zinc tablets (*p* = 0.000), child provided with deworming tablets (*p* = 0.000), child promoted to eat fortified food (*p* = 0.000), child's anthropometric measurements were taken (*p* = 0.000) as seen in Table 4.

**Table 4:** Details of child healthcare services availed by mothers stratified with their COVID-19 status during November 2021- October 2022 in Ahmedabad, India

Child Health Services	Total *N* = 253 (%)	COVID -ve mothers *n* = 153 (%)	COVID + ve mothers *n* = 100 (%)	*p-value*
Vit K inj. after birth	229 (90.5)	142 (92.8)	87 (87)	0.001
COVID-19 test after birth	68 (26.9)	27 (17.7)	41 (41)	0.000
Test for abnormalities/ congenital defects	18 (7.1)	13 (8.5)	5 (5)	0.001
IFA tablets	137 (54.2)	93 (60.8)	44 (44)	0.000
ORS drink	137 (54.2)	93 (60.8)	44 (44)	0.000
Zinc tablets	121(47.8)	85 (56.2)	35 (35)	0.000
Deworming tablets	114 (45.1)	78 (51)	36 (36)	0.000
Fortified food	192 (75.9)	130 (84.9)	62 (62)	0.000
Record of anthropometrics	166 (65.7)	131 (85.7)	35 (35)	0.000

*IFA* Iron and Folic Acid, *ORS* Oral Rehydration Solution, *AWW* Anganwadi Worker, *HW* Health worker

Regression analysis indicated that women without COVID-19 had significantly higher odds of availing maternal and child healthcare services as compared to COVID-19-positive women. COVID-19-negative were substantially more likely to register their pregnancies with healthcare workers compared to COVID-19-positive women [aOR = 9.02 (1.75–46.37)]. Similarly, they are likely to receive home visits from healthcare workers [aOR = 2.59 (1.03- 6.49)] and receive counselling related to increased consumption of iron-rich food sources [aOR = 1.92 (0.61- 6.06)]. Similarly women without COVID-19 were more likely to discuss or prepare a birth plan with healthcare workers [aOR = 2.54 (1.12- 5.82)], and they were more likely to plan their place of delivery and deliver at that planned location ([aOR = 1.98 (0.99- 3.92)]. Women without COVID-19 were more likely to be accompanied by healthcare workers during labor ([aOR = 2.91(1.04- 8.11) and to receive appropriate birth spacing counseling [aOR = 1.38 (0.7–2.71)]. Most child healthcare services remained unaltered for both types of COVID-19 mothers, except to receive deworming tablets [aOR = 2.51 (1.06–5.95)]. This indicates that the child healthcare services are not much differentiated with respect to the mother’s COVID-19 status. The details are shown in Table 5.

**Table 5:** Women without COVID-19 had better healthcare services as compared to the COVID-19-positive women during November 2021- October 2022 in Ahmedabad, India

Healthcare services	Crude OR (95% CI)	Adjusted OR (95%CI)
**Maternal healthcare (ANC) services**		
Registration of pregnancy by healthcare workers	11.92 (4.052–35.08)	9.02 (1.75–46.37)
Received Mother and Child Protection Card	7.48 (2.97- 18.8)	1.76 (0.45- 6.95)
Health care workers like ASHA, ANM/ MPHW visited their doorstep	5.63 (2.94- 10.77)	2.59 (1.03- 6.49)
Made aware about dates for next antenatal visits and EDD	3.01 (1.35–6.70)	0.35 (0.09- 1.34)
Received medicines of iron-folic acid and calcium	2.49 (1.27- 4.9)	0.80 (0.28- 2.28)
Received counselling related to increase consumption of iron rich food source	2.43 (1.21–4.86)	1.92 (0.61- 6.06)
Discuss/ prepare birth plan for your pregnancy by any HW	4.01 (2.37–6.79)	2.54 (1.12- 5.82)
Enrolled for any government scheme like JSY, JSSK, KPSY, PMSMA or SUMAN	1.47 (0.782- 2.76)	0.93 (0.44- 1.97)
**Maternal healthcare (INC/PNC) services**		
Same planned place of delivery and actual place of delivery	3.44 (1.99–5.95)	1.98 (0.99- 3.92)
Accompany you during your labor	2.48 (1.09–5.64)	2.91(1.04- 8.11)
Received any counselling related to birth spacing/family planning	2.09 (1.27- 3.46)	1.38 (0.7–2.71)
**Child healthcare services**		
Baby received Vitamin K injection immediately after birth	1.89 (0.81–4.41)	1.19 (0.339–3.66)
Child tested for other abnormality/congenital defects	1.79 (0.62- 5.20)	0.76 (0.15- 3.74)
Immunization received as per age of the child	1.57 (0.83- 2.97)	1.2 (0.88–4.22)
Child provided IFA tablets/bottle	1.90 (1.146- 3.17)	0.79 (0.255- 2.45)
Child provided ORS packets	2.12 (1.26- 3.56)	0.48 (0.098- 2.38)
Child provided Zinc tablets	2.42 (1.44- 4.09)	1.82 (0.34–10.08)
Child provided with deworming tablet/bottle	3.39 (1.86- 6.16)	2.51 (1.06–5.95)

### Qualitative findings

#### Health system determinant

##### Service delivery

The respondents reported that the pandemic caused issues in availing of the services irrespective of whether the women were COVID-positive or not. Most women reported that a healthcare worker did not visit them for ANC or PNC, which is contrary to the survey findings. Most women went to private or government hospitals for their ANC and PNC visits. The unavailability of doctors was also one issue, and some had to shift to another hospital for delivery during labour. There were also instances where the women changed the hospital because of fear of contracting infection in the government hospital.

"*My family and myself everyone were anxious because it was my first delivery. The public hospital doctor tested positive for corona; so there was no doctor to do my delivery then, we shifted to a private hospital…….".-A COVID-negative mother*

The women reported that the doctors’ assurance of their availability and continued telephonic support provided by the medical officers and private providers reduced their worries. They also mentioned that their doctors tried to provide as much support as possible. On the other hand, some believed they could have received better ANC and PNC services if no pandemic had existed. The women, irrespective of their COVID-19 infection status, worried about their child's safety during and after delivery at the hospital. One COVID-positive woman during the delivery complained about the negligence of newborn care when her child became sick.

"*Had no fear because doctors told us they are available',' doctor was available every time I wanted to speak to him, so was less worried" – COVID-positive mother.*

"….. after delivery, they didn't check my baby and discharged the baby after reaching home the baby almost died…….". – COVID-positive mother

"The government facility was better in comparison to private….". – COVID-positive mother

##### Enrollment in the schemes

The women were usually enrolled in government schemes, but some mentioned that they had not received any monetary benefits. Few of them did not register for the schemes. Everyone had been issued the 'Mamta card' (Mother and Child Protection card- Tool for families to learn, understand and follow positive practices for achieving good health of pregnant women, young mothers, and children.), and was aware of the 'Mamata Divas' (Village Health & Nutrition day where a special focus is given on the child healthcare services and specifically immunization services in the particular area of village or city) in their areas. The ASHA workers visited them but were unsure about the benefits the mothers were entitled to receive from the government.

"*Kaki would visit me sometimes but no one else came to visit me ….."- A COVID-negative mother.*

#### Social determinant

##### Fear of contagion

Fear of infection was widespread among the mothers. Women who were frontline workers mentioned families requesting them to leave their jobs during pregnancy for fear of contracting a COVID infection or themselves being worried about illness while at work during the pandemic.

"*My mother-in-law asked me to quit my job, said child and my health are more important than my job, But I love my job…." – A COVID-positive mother.*

*"……I still continued my job while I was pregnant, but there was fear in the back of my mind". – A COVID-positive mother*

*"…………by seeing the news, I was anxious because that time children used to get affected by positive mother…."* *. – A COVID-positive mother*

*"………..went for injection of minimum blood loss during delivery and then tested positive". –A COVID-positive mother*

## Discussion

The COVID-19 pandemic posed a challenge to healthcare utilisation, but there was not much variation regarding access to ANC, INC, or PNC services. A modelling study on the indirect effects of the pandemic in 118 LMIC estimated a reduction in ANC by at least 18%, and possibly up to 51.9% [17] whereas in our study, it was seen that there had been an increase in access to ANC and PNC services in Ahmedabad may be because of preparedness of the system, and support of other departments in providing uninterrupted health services during the pandemic. Another reason for the difference is also the social stigma, where there are certain panics among the pregnant women during the pandemic. The various experiences like unavailability of a doctor due to him being tested positive for COVID–19 or fear of infection were the most cited barriers to utilising maternal and childcare services in Ahmedabad was similar to experiences documented in Uttar Pradesh [18]. The women who tested positive for COVID-19 utilized less child healthcare services as compared to women who did not test positive. The testing for abnormalities or congenital anomalies among the newborns was very low whereas covid testing, provision of ORS packets, Zinc supplements, IFA supplements, and Vitamin K injection was comparatively higher among both mothers who tested positive for covid-19 infection as well as mothers who didn’t test positive for covid-19 infection. The fear of the effect of infection on the fetus or child could be a driving factor for women to seek ANC and PNC services, whereas fear of contagion is a barrier to accessing maternal and child healthcare services.

In our study it was also seen that Multiparous women availed the maternal and child healthcare services more than nulliparous women; this could be due to their prior experience and understanding of the need for ANC services. Same thing was also observed in the study from Malaysia where multiparous women availed more services as they were more aware and informed about the benefits [19], but it is opposite to studies in Pakistan and Ghana where nulliparous women availed ANC services more [20, 21]. In a study in Malaysia, women who were less educated availed lesser services compared to women who were more educated [19]. This is similar to what was seen in Ahmedabad, where women who are more educated availed more services. Similarly, in Northeast Ethiopia, people with no formal education were less likely to utilise ANC services [22] whereas in Ghana, there was no significant relationship between education and utilisation of maternal and child healthcare services [21]. In Malaysia, middle-aged women or women between the age group of 20–34 availed more services than women below 19 or above 35 years of age [19], similar to Ahmedabad, where women below the age of 31 years availed more services compared to women above this age. In contrast to these findings, mothers in Northeast Ethiopia above the age of 35 years utilised ANC services more [22].

Fear of infection, the transmission of infection to the child, and the facility not being functional are some of the reasons for missing appointments, which is similar to the results of the study from Saudi Arabia, where the most common reasons for missing appointments in primary care and hospitals, respectively, were: fear of infection 52% and 47%, the facility not working as usual 25% and 7.5%, fear of infection to a child 19% and 17% [23].

The diversion of maternal care services, like a change of place for the delivery or a change of ANC check-up due to fear of COVID-19 infection, was similar to the diversion reported in Northeast Ethiopia [22]. The two women who had a delivery at home assisted by untrained personnel in Ahmedabad indicated the prevalence of home delivery here. This also indicates that more intensive measures have to be taken to ensure 100% institutional deliveries by trained health workers.

The utilisation of maternal care services documented through this research is comparable with National Family Health Survey-5 findings. According to the NFHS 5 [24], zones 8% of women had more than 3 ANC visits, 98% received mother and child protection cards, 89% received Iron and folic acid supplements, 92% received counselling on family planning, 95% had institutional deliveries, and 93% were assisted by health personnel whereas compared to NFHS 5 data, the research indicates 97.1% had more than 3 ANC visits, 88.1% received mother and child protection cards, 84.9% received Iron and folic acid supplements, 99% of women had institutional deliveries and had their delivery assisted by health care workers. Thus, the ANC checkups and institutional deliveries increased during the pandemic, but other services like issuing of mother and child protection card, and distribution of Iron and folic acid supplements decreased during the pandemic.

This study has several limitations due to the selection process, design, and time point. First, the study has recruited participants from a few zones of the city based on administrative permissions and feasibility: therefore, the generalisation of the findings need to be carefully interpreted. Second, although the study sampling attempted to balance the place of delivery and the COVID status, a system-side quality check was not within the scope of the study, therefore the type of private settings and its quality were not documented. Third, the women might have recall bias due to a difference in the time of delivery and the time of data collection, as the data was collected during the post-pandemic period.

## Conclusion

Overall, health services were disrupted during the pandemic. However, the utilisation of ANC and PNC services was less affected and differed for women who didn’t test positive for COVID-19 during their pregnancy in comparison to those who did. As the interviews showed, pregnant women tended to prefer private facilities to large government hospitals. This is even significantly higher with the positive-tested pregnant women. Fear of infection could be the reason for negative-tested women to avoid large governmental hospitals. For women who tested positive, the function of smaller private facilities as COVID-19 designated centres might have influenced this preference.

Rare cases of home deliveries by unskilled attendants, the lower rate of post-natal services for babies of positive-tested mothers, and lacking awareness of entitlements to public subsidies show a need for improvement. The doctors' assurance played a crucial role in providing psychological support to the women during their pregnancy; it also helped decrease the anxiety of women and their families. This was highly needed as women felt confident to go for deliveries at public hospitals, e.g. as frontline workers, and had fears of a negative impact on their children’s health there by they opted for deliveries at private facilities.

Thus, these findings show the gap in access to health care services as well as the difference in utilization of services among pregnant women who tested positive for COVID-19 in comparison to those who did not and highlight the need for a resilient health system and health services so that essential services like maternal and child healthcare services are not compromised during this kind of crises. Thus, planning resilient healthcare will be helpful in managing any future outbreaks.

## Data Availability

Data from this study will be available at the Center for One Health Education, Research & Development (COEHRD), Indian Institute of Public Health Gandhinagar, India, after the completion of this study. Researchers who meet the criteria for access to confidential data are encouraged to approach the corresponding author for the same.
